# A Gestalt inference model for auditory scene segregation

**DOI:** 10.1371/journal.pcbi.1006711

**Published:** 2019-01-22

**Authors:** Debmalya Chakrabarty, Mounya Elhilali

**Affiliations:** Laboratory for Computational Audio Processing, Center for Speech and Language Processing, Department of Electrical and Computer Engineering, Johns Hopkins University, Baltimore, MD, USA; University of California at Berkeley, UNITED STATES

## Abstract

Our current understanding of how the brain segregates auditory scenes into meaningful objects is in line with a Gestaltism framework. These Gestalt principles suggest a theory of how different attributes of the soundscape are extracted then bound together into separate groups that reflect different objects or streams present in the scene. These cues are thought to reflect the underlying statistical structure of natural sounds in a similar way that statistics of natural images are closely linked to the principles that guide figure-ground segregation and object segmentation in vision. In the present study, we leverage inference in stochastic neural networks to learn emergent grouping cues directly from natural soundscapes including speech, music and sounds in nature. The model learns a hierarchy of local and global spectro-temporal attributes reminiscent of simultaneous and sequential Gestalt cues that underlie the organization of auditory scenes. These mappings operate at multiple time scales to analyze an incoming complex scene and are then fused using a Hebbian network that binds together coherent features into perceptually-segregated auditory objects. The proposed architecture successfully emulates a wide range of well established auditory scene segregation phenomena and quantifies the complimentary role of segregation and binding cues in driving auditory scene segregation.

## Author summary

In every day life, our brain is able to effortlessly make sense of the cacophony of sounds that constantly enter our ears and organize them into meaningful sound objects. In this work, we use an architecture based on stochastic neural networks to ‘learn’ from natural sounds which cues are crucial to the process of auditory scene organization. The computational model delivers a hierarchical architecture that mimics multistage processing in the biological auditory system. It learns a rich hierarchy of spectral and temporal features that allow the decomposition of an auditory scene into informative components. These features are then grouped together into coherent objects based on Hebbian learning principles. Though trained on unrelated datasets of natural sounds, the model is able to replicate human perception of auditory scenes in a wide variety of soundscapes ranging from simple tone sequences to complex speech-in-noise scenes.

## Introduction

We live in busy environments, and our surrounds continuously flood our sensory system with complex information that needs to be analyzed in order to make sense of the world around us. This process, labeled scene analysis, is common across all sensory modalities including vision, audition and olfaction [1]. It refers to the ability of humans, animals and machines alike to parse the mixture of cues impinging on our senses, organize them into meaningful groups and map them onto relevant foreground and background objects. Our brain relies on innate dispositions that aid this process and help guide the organization of patterns into perceived objects [2]. These dispositions, referred to as Gestalt principles, inform our current understanding of the perceptual organization of scenes [3, 4].

In most theoretical accounts, the role of Gestalt principles in parsing a scene can be conceptualized in two stages: segregation (or analysis) and grouping (or fusion) [5]. In the first stage, the sensory mixture is decomposed into feature elements, believed to be the building blocks of the scene. These features reflect the physical nature of sources in the scene, the state and structure of the environment itself, as well as perceptual mappings of these attributes as viewed by the sensory system. These features vary in complexity along a continuum from basic attributes (e.g. edges or frequency components) to more complex characteristics of the scene (e.g. shapes or timbral profiles). The ubiquitous nature of these profiles often conceals the multiplexed structures that underlie this analysis of scene features in the brain. In most computational accounts, this segregation stage is modeled using feature analyses which map the sensory signal into its building blocks ranging from simple components (e.g. frequency channels) to dimensionally-complex kernels [6, 7].

Processing the distinctive features of a scene is generally followed by a fusion stage which integrates the state and behavior of the scene’s building blocks using grouping mechanisms that reflect the local and global distribution and dynamics of the features. This stage employs ‘rules’ that guide how grouped elements give rise to perceptually coherent structures forming *objects* or *streams* [2, 8, 9]. In many mathematical models, these grouping cues are often leveraged in back-end classifiers that are tuned to capture patterns and relationships within specific object classes (e.g. speech, music, faces, etc) [10–13]. In doing so, these models effectively capture the inter-dependencies between object attributes and learn their mapping onto an integrated representational space [14–16]. Ultimately, success in tackling scene analysis depends on two key components [17]: (i) obtaining a rich and robust feature representation that can capture object specific details present in the scene; (ii) grouping the feature elements such that their spatial and temporal associations match the dynamics of objects within the scene.

Vision models have been very successful in mining these two aspects of scene analysis. Intricate hierarchical systems have leveraged inherent structure in static and dynamic images to extract increasingly elaborate features from a scene that are then used to segment it, interpret its objects or track them over time [18–20]. Data-driven approaches have shown that high dimensional feature spaces are very effective in extracting meaningful semantics from arbitrary natural images [20–22]; while hand-engineered features like scale-invariant feature transform (SIFT) [23], histogram of oriented gradients (HOG) [24], and Bag-of-visual-word descriptor [25] among others have also enjoyed a great deal of success in tackling computer vision problems like image classification and object detection. Recent advances in deep layered architectures have resulted in a flurry of rich representational spaces showing selectivity to contours, corners, angles and surface boundaries in images [26–29]. The deep nature of these architectures has also led to a natural evolution from low-level features to more complex, higher-level embeddings that capture scene semantics or syntax [30, 31].

In audition, computational approaches to tackle auditory scene organization have mostly taken advantage of physiological and perceptual underpinnings of sound processing [17]. A large body of work has built on knowledge of the auditory pathway, particularly the peripheral system to build sophisticated analysis models of auditory scenes. These systems extract relevant cues from a scene, such as its spectral content, spatial structure as well as temporal dynamics; hence allowing sound events with uncorrelated acoustic behavior to occupy different subspaces in the analysis stage. These models are quite effective in replicating perceptual results of stream segregation especially using simple tone and noise stimuli [32–37]. Some models also extend beyond early acoustic features to examine feature binding mechanisms that can be used as an effective strategy in segregating wide range of stimuli from simple tone sequences to spectro-temporally complex sounds like speech and music [38–40]. In most approaches however, the models are built around hand-crafted feature representations, hence limiting their scope to specific mappings of the acoustic space. With the emergence of deep belief architectures, recent efforts started learning rich feature spaces from natural soundscapes in a data driven fashion, and subsequently using these spaces in domains like music genre classification, phoneme classification and speaker identification [41–44]. Applications of deep learning have also successfully tackled the problem of speech separation even with monaural inputs by learning embeddings of a speaker’s time-frequency dynamics against other speakers [45, 46].

The current study also leverages neural network theory to ‘learn’ Gestalt principles directly from sound. The work examines what kind of cues can one *infer* from natural sounds; how well do these learned cue reflect the known Gestalt components of auditory streams; and how effective are these cues in explaining perceptual organization of auditory scenes with varying degrees of complexity. The model is devised as a hierarchical structure that generally follows the two-stage pipeline of analysis then fusion, in line with prototypical scene analysis theories [5]. This system analyzes the incoming acoustic signal with a multitude of granularities, hence allowing both local and global acoustic attributes to emerge. The short-term analysis performs a local tiling of the spectro-temporal space; hence inferring *simultaneous* grouping cues [47–49]. A longer-range analysis extends the segregation stage to examine temporal dependencies across acoustic attributes over different time scales; hence exploring emergence of *sequential* grouping cues [50–54]. Finally, a fusion stage binds the cues together based on how strongly they correlate with each other across multiple time scales. This integration is achieved using *Hebbian* learning which reinforces activity across coherent channels and suppresses activity across incoherent ones [55–57]. Apart from the basic layout and choice of analysis window sizes, the network is trained in an unsupervised fashion on a rich sound dataset including speech and nature sounds hence offering a general inference architecture of auditory Gestalt cues that are common across many sound environments.

The overall system is tested with a wide range of stimuli where we can quantify the role of each and every component of the network in driving stream segregation processes. We also contrast the system performance with a set of control experiments where different components of the model are deliberately switched on/off in order to examine their impact on the organization of different acoustic scenes. These control experiments aim not only to dissect the role of various system components. They also shed light on how necessary and/or sufficient different grouping cues are to anchor the analysis of different stimuli structures and sound types. The paper first presents an in-depth description of the proposed architecture, followed by an analysis of the emergent properties of the trained network and their potential neural correlates in the auditory pathway. The experimental results outline how the network replicates human psychoacoustic behavior in stream segregation and speech intelligibility paradigms. Finally, we present control experiments that dissect the network architecture and examine the contribution its component. We discuss the implications of this network in shedding light on ties between observed perceptual performance in various complex auditory scenes and the neural underpinnings of this behavior as implemented in networks of neurons along the auditory pathway.

## Results

### A Gestalt inference model for auditory scene segregation

A number of Gestalt principles have been posited as indispensable anchors used by the brain to guide the segregation of auditory scenes into perceptually meaningful objects [8, 47, 58]. These comprise a wide variety of cues; for instance harmonicity which couples harmonically-related frequency channels together, common fate which favors sound elements that co-vary in amplitude, and common onsets which groups components that share a similar starting time and to a lesser degree a common ending time. Most of these cues are thought to be innate in our auditory system, and evidence for their role is found across many species [59–63]. These processes likely take advantage of statistical regularities of sounds in natural environments and reflect the physical constraints of sound generation and propagation (e.g. two sound sources rarely start at the exactly the same time; periodic vibrations induce resonant modes at integer multiples of the fundamental frequency). Here, we examine whether a statistical inference model can learn these cues directly from natural sounds; and if so, how effective are these learned cues relative to existing hand-tailored segregation systems.

The proposed model is designed as a hierarchical system that explicitly mimics an ‘analysis-then-fusion’ processing pipeline. The analysis stage is itself laid out in two stages. First, an analysis of local spectrotemporal cues aims to learn *simultaneous* Gestalt cues believed to operate over short-time scales in order to locally segregate sound elements. Second, an analysis of more global cues operates over longer time-scales and aims to learn *sequential* Gestalt cues that enable tracking dynamics of elements from the first stage at a temporal or melodic level [8]. Following these stages is a fusion step that combines together segregated elements that constitute different auditory objects, using principles of *temporal coherence* [39, 64, 65]. The Gestalt analysis stages are learned directly from natural sounds in a generative fashion, allowing each component of the model to represent natural sounds from its own vantage point following principles of stochastic neural networks, as detailed next. The fusion stage merely organizes or fuses these learned patterns following the concept of temporal coherence, as also detailed later.

**Fig 1:**
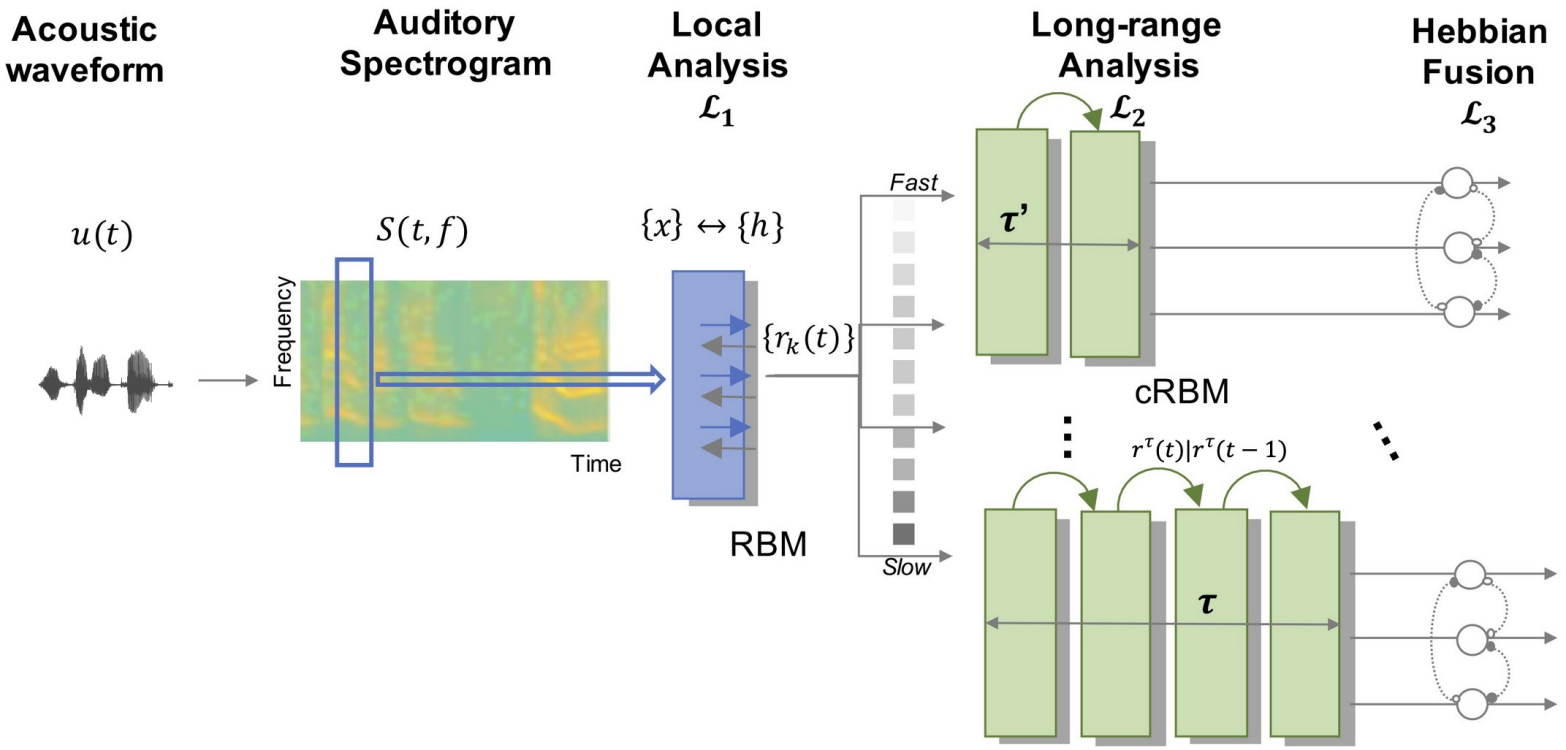
Schematic of the proposed model. An acoustic signal *u*(*t*) undergoes a series of transformations starting with a mapping to a time-frequency spectrogram, followed by two-layers of stochastic neural networks (local analysis $L_{1}$ and long-range analysis $L_{2}$), then a fusion stage $L_{3}$.

Fig 1 depicts a schematic of the overall model. It takes as input the acoustic waveform of an auditory scene *u*(*t*) and maps it onto a time-frequency representation, using a biomimetic peripheral model from Yang *et al*. [66]. Briefly, this transformation analyzes the acoustic signal *u*(*t*) using a bank of logarithmically-spaced cochlear filters whose outputs are further sharpened via a first order derivative along the frequency axis, followed by half wave rectification and short term integration over 10ms frames (see Methods for details). This filterbank analysis results in an auditory spectrogram represented by *S*(*t*, *f*).

The following stage (called $L_{1}$) is structured as a two-layer sparse Restricted Boltzmann Machine (sparse RBM) with a fully connected visible and hidden layer [67]. It takes as input 3 consecutive frames of the spectrogram and learns a probability distribution over the set of these short tokens. RBMs are powerful stochastic neural networks that are conceptually similar to autoencoders but can infer statistical distributions over their input set [68]. A RBM layer is chosen for this stage in order to explore the space of local spectrotemporal tokens and learn latent cues that represent statistical structures in natural sounds over short time scales. The visible layer units {*x*_*k*_} are real-valued and characterized by a Gaussian distribution fitted over the input spectrogram *S*(*t*, *f*); while hidden units {*h*_*k*_} are sampled from a Bernoulli distribution for *k* = 1, 2, …, *K* where *K* is the number of nodes in each layer. The network is parameterized by Θ = {*W*, *A*, *B*} where *W* represents the interconnected weights between visible and hidden units, and *A* (*B*) represents the visible (hidden) bias, respectively. The network is trained using a Contrastive Divergence (CD) algorithm with the objective to minimize the reconstruction error between *x* and $\hat{x}=hW+A$ [69].

By learning the regularities in local spectrotemporal tokens of natural sounds, the connection weights *W* effectively span an array of latent cues that reflect the structure of soundscapes. Our hypothesis is that these latent factors represent the so-called simultaneous cues used as Gestalt principles for sound analysis. After training, connection weights are transformed into a 2D filter F(t,f), akin to spectro-temporal receptive fields derived from neural activity of biological neurons in the auditory system [70]. These learned filters are then applied in a convolutional fashion over the incoming spectrogram *S*(*t*, *f*) to derive the outputs of layer $L_{1}$ nodes. These responses are further subjected to a neural adaptation stage which imposes a dynamic regulation of the response of each filter hence suppressing units with weak activation (see Methods for details).

$L_{1}$ responses are then processed by the next layer in the model which completes the analysis stage to infer possible sequential cues that extend over longer time constants. This second layer $L_{2}$ is devised as an array of conditional RBMs (cRBMs), which are extended versions of RBMs designed to model temporal dependencies [71]. Similar to a RBM, a cRBM consists of a visible layer with units {*x*_*k*_}, assumed to arise from a Gaussian distribution fitted over the input, and a hidden layer with {*h*_*k*_} units sampled from a Bernoulli distribution. Unlike a RBM, a cRBM acts as a dynamical system operating over an entire input history *τ* taking as input occurrences at times {*t*, *t* − 1, …, *t* − *τ*} in order to capture dynamics in the input space over context *τ*. In the current model, we explore sequential cues over a range of temporal contexts and construct an array of parallel cRBM networks over multiple histories ranging in temporal resolutions from *τ* ∼ (30–600 *ms*). $L_{2}$ is parameterized by Θ = {*W*, *A*^*τ*^, *B*^*τ*^, *C*^*τ*^, *D*^*τ*^} where *W* represents the interconnected weights between visible and hidden units and capture the interactions across input features over an extended temporal history *τ*, *A*^*τ*^ and *B*^*τ*^ represent the visible and hidden biases, respectively, while *C*^*τ*^ and *D*^*τ*^ quantify autoregressive weights between past inputs and the current input (or current hidden unit, respectively). Just like the localized layer $L_{1}$, the contextual layer $L_{2}$ is trained in a generative fashion using contrastive divergence (CD) in order to best capture the dynamics in natural sounds using the same dataset of realistic sounds spanning speech, music and natural sounds. Here again, our hypothesis is that the stochastic cRBM learns latent parameters Θ that reflect the sequential cues underlying dynamics of natural sounds over a wide range of temporal contexts. Once trained, the model parameters are applied to incoming $L_{1}$ filter responses in a linear fashion, yielding a multi-resolution output which is then passed over to the next stage in the hierarchy (see Methods for details).

The next layer in the hierarchy focuses on a fusion operation to facilitate the grouping of perceptually-coherent objects. This binding stage explores co-activations across all $L_{2}$ channels within a given context *τ* and binds together the units that exhibit strong temporal coherence [64, 72]. The *‘temporal coherence’* theory posits that emergence of perceptual representations of auditory objects depends upon *strong* coherence across cues emanating from same object and *weaker* co-activation across cues from competing objects. This coherence is not an instantaneous correlation but one that is accumulated over longer time scales, commensurate with the contextual windows explored in the $L_{2}$ layer. We implement this concept in a biologically-plausible fashion via mechanisms of Hebbian learning, which suggests that when two neurons fire together, their synaptic connection gets stronger [73]. Effectively, Hebbian interactions operate by reinforcing activity across coherent channels, hence grouping them into putative objects and inhibiting activity across incoherent channels [74]. We implement a synaptic interaction across output channels from layer $L_{2}$ by introducing a coherence synaptic weight matrix *V*. If two units *i* and *j* are co-activated at a given time *t*, their corresponding synaptic connection *V*_*ij*_ is reinforced over time. If the correlation between their activity is weak, the corresponding synaptic weight *V*_*ij*_ is reduced accordingly. These synaptic weights are applied to the output of each channel in a dynamic fashion, hence modulating the activity across an entire ensemble of neurons within each context in layer $L_{2}$. The net effect gives emergence to perceptual coherent groups that represent auditory objects in a scene. A final read-out stage is then appended to the model to extract responses to different stimuli and test the degree of segregation of different objects, as viewed by the model outputs (see Methods for details).

### Model characterization

In order to examine the emergent sensitivity of learned layers in the network, we derive the tuning characteristics of individual nodes or neurons and explore their filtering properties in the modulation domain [75, 76]. Modulation tuning reflects stimulus cues that best drive individual nodes in the model both in terms of temporal variations and dynamics (i.e. temporal modulations or rates) as well as spectral span and bandwidth (i.e. spectral modulations or scales). This approach follows common empirical techniques used in electrophyisology and psychophysics to probe the tuning of a system to specific acoustic cues. It is specifically used to characterize spectro-temporal receptive fields (STRFs) which offer 2-dimensional profiles of filtering characteristics of neurons [70].

**Fig 2:**
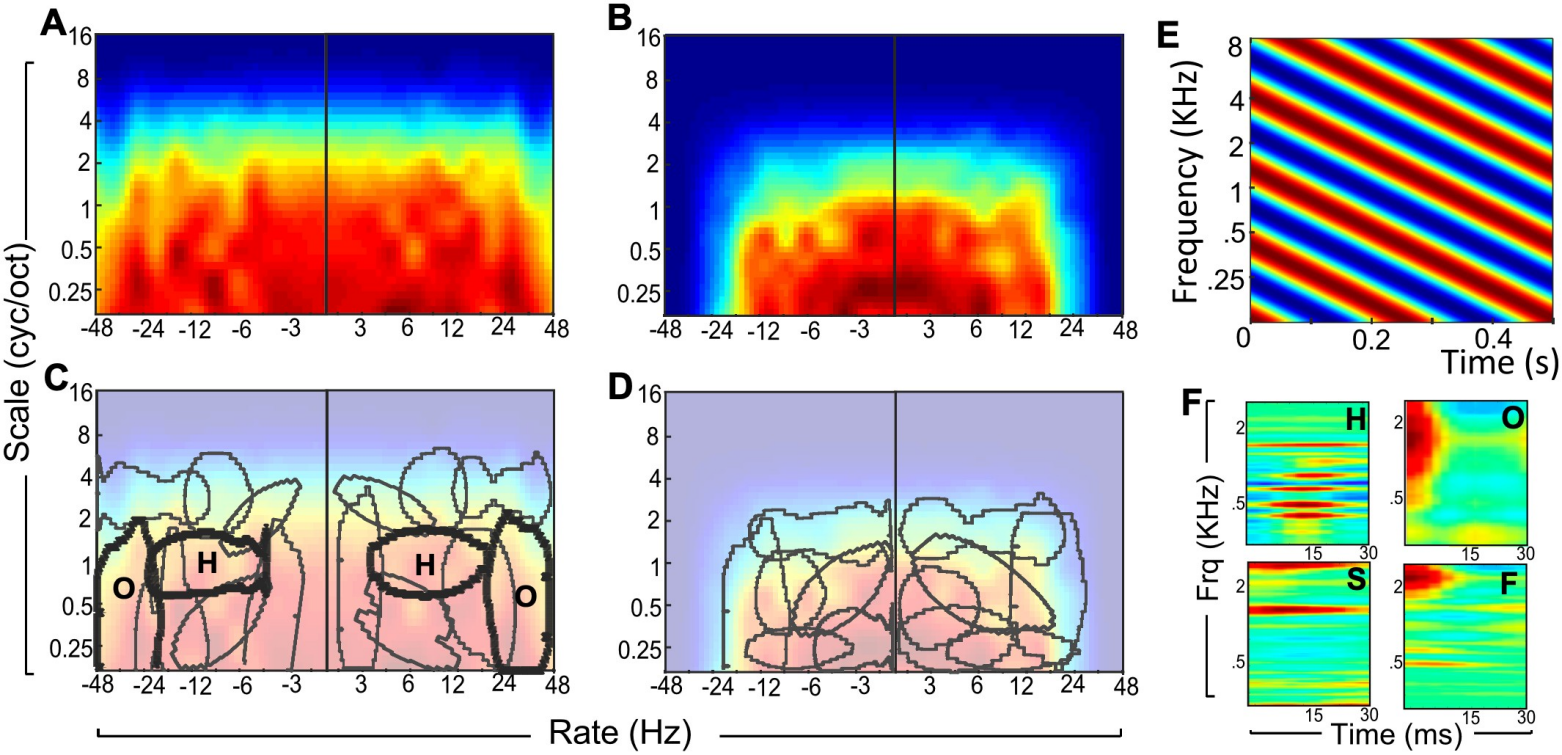
Modulation characteristics of the network. (A,B) Normalized modulation transfer function for layers $L_{1}$ -left- and $L_{2}$ -right- displayed in axes of rate (temporal modulations in Hz)—scale (spectral modulations in cycles per octave). (C,D) Overlaid on each transfer function is a contour plot of agglomerative clusters in spectro-temporal modulation space for layers $L_{1}$ -left- and $L_{2}$ -right-.(E) Noise ripples are used to analyze the spectro-temporal tuning of the model at different stages. They are noise signals that are modulated in time and frequency. (F) Example filter tuning F(t,f) from layer $L_{1}$ for 4 nodes exhibiting tuning to harmonicity (top, left), onset (top, right), a slow neuron (bottom, left) and a fast neuron (bottom, right). The filter response profiles have been interpolated using a cubic function for display purposes.

First, we employ a classic transfer function method using probe stimuli in order to derive the tuning of both $L_{1}$ and $L_{2}$ layers of the network [77–79]. We present modulated noise signals (called ripples) as input to the model with varying spectro-temporal modulation parameters (Fig 2E) and characterize the fidelity of the ripple encoding at various stages of the network as the ripple modulation parameters are varied [80]. Each ripple is constructed as a broadband noise signal whose envelope is modulated both in time and frequency, with temporal modulation parameter *ω* (in Hz) and spectral modulation parameter Ω (in cyc/oct) (see Methods for details).

By sweeping through a range of ripple parameters, we compute a normalized modulation transfer function (MTF) from the response of layers $L_{1}$ and $L_{2}$ which quantifies the synchronized response of each layer to the corresponding dynamics in the ripple stimulus (see Methods for details). $L_{3}$ is not a trained layer and hence is not subject to this analysis. Fig 2A and 2B depict the MTF derived from both $L_{1}$ and $L_{2}$. The functions highlight that both layers exhibit a general low-pass behavior both along temporal and spectral modulations. As expected, layer $L_{1}$ is trained over shorter time-scales and does exhibit faster temporal dynamics along the rate axis, while the contextual layer $L_{2}$ is mostly tuned to slower dynamics < 30*Hz* with a slightly tighter spectral selectivity mostly concentrated below 1 cycles/oct. This outcome is very reminiscent of similar transfer functions obtained from neurophysiological data showing contrasting tuning characterizations in the midbrain, auditory thalamus and auditory cortex [81–83], whereby selectivity of individual neurons along the mammalian auditory hierarchy evolves from faster to slower temporal dynamics and from more refined to broader spectral spans along frequency.

We further examine the selectivity of *individual* neurons and compare emergent tuning characteristics common across nodes in the network by employing an agglomerative clustering algorithm (see Methods for details). This approach clusters nodes exhibiting similar tuning profiles into common groups hence providing insight into the underlying acoustic cues being processed by each cluster. Fig 2C and 2D show contour plots from the resulting clusters overlaid on the MTF profiles for layers $L_{1}$ and $L_{2}$. The array of clusters indicates that neurons in each of these layers do indeed exhibit a wide variety of selectivity to spectral and temporal dynamics in the input signal. We specifically note a cluster of $L_{1}$ neurons that is more sensitive to fast transients or ‘onsets’. This group is labeled ‘O’ in Fig 2C. An example time-frequency profile F(t,f) of a neuron in the ‘O’ cluster is shown in Fig 2F (upper-right). We also note a spectrally-structured cluster (labeled ‘H’) centered around spectral modulations ∈ [1-2] cyc/oct corresponding to harmonic peaks present in natural sounds. An example neuron from this cluster is shown in Fig 2F (upper-left) and highlights the selectivity to specific frequency bands in the input spectrogram. The clustering procedure also reveals the presence of oriented spectro-temporally selective clusters, likely tuned to detect frequency-modulated sweeps in the signal over different spectrotemporal scales; as well as other clusters with special selectivity to spectral or temporal features. Fig 2F (lower panels) shows an example of two $L_{1}$ neurons with different temporal dynamics contrasting a slow neuron ‘S’ and a fast neuron ‘F’.

### Stream segregation experiments

**Fig 3:**
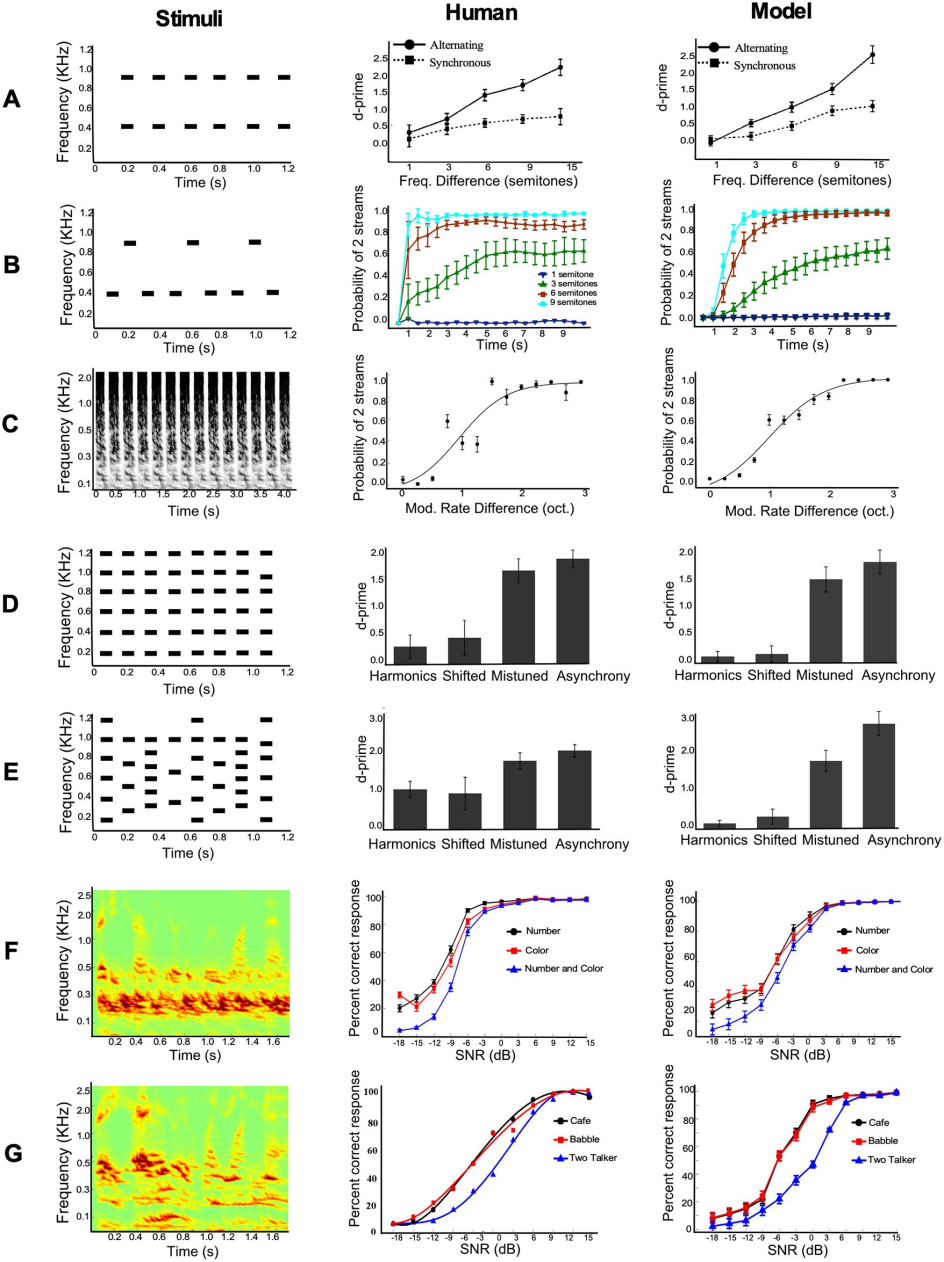
Primary results of stream segregation using proposed model. Leftmost panel shows the stimuli sequence used for each experiment. Middle panel shows human listening performance and rightmost panel shows the model performance. Row (A) replicates experiments from [86], row (B) replicates experiments from [90], row (C) replicates experiments from [92], rows (D) and (E) replicate experiments from [93], and rows (F) and (G) replicate experiments from [95, 96].

We test the model’s behavior with a variety of acoustic scenes ranging from classic streaming paradigms using simple tones to experiments using speech signals. Crucially, all experiments are tested on the *same model* (after all layers have been trained), without any adjustment to model parameters. The stimulus parameters are carefully chosen to closely replicate previously published human perceptual experiments hence allowing a direct comparison between the model and human perception. All stream segregation results are shown in Fig 3 organized in 3 columns: the stimulus on the left, a replica of human perception of the same stimulus reproduced from the corresponding publication in the center, and the model performance on the right.

#### Simple tones

The first experiment employs the classic two-tone paradigm with sequences of high and low notes, commonly used in streaming experiments [8, 84, 85]. The sequences are produced by presenting two tones of different frequencies, *A* and *B*, repeatedly and in alternation (*ABAB*−). When the frequency separation Δ*F* between the *A* and *B* tones is relatively small (<10%), listeners perceive the sequence as grouped or fused and report hearing one stream. As the frequency separation Δ*F* increases, listeners hear two separate streams consisting of only low notes (*A* − *A* −) or only high notes (−*B* − *B*-). In contrast, when the two *A* and *B* notes are presented synchronously (Fig 3A-left), listeners tend to hear the sequence as grouped regardless of the frequency separation Δ*F*, in a process reminiscent of temporal coherence which fuses together channels that are co-activated together [64, 72]. Fig 3A-middle replicates results from a study by Micheyl *et al*. [86]. The study shows that an alternating tone sequence is perceived as a single stream when the frequency separation Δ*F* is small and is segregated into two streams when Δ*F* is large. When the two tones are presented synchronously, they are always perceived as grouped regardless of frequency separation. The fused percept is objectively measured using d’ [87, 88]; where listeners are asked to detect a change in one of the tones presented in the final burst (see Methods for details). Fig 3A-right shows that the model replicates the same behavior using the same tone sequences presented in alternation or synchrony. As the frequency separation Δ*F* increases between the *A* and *B* tones, the model is more likely to perceive them as segregated in the alternating condition but tends to fuse them in the synchronous condition.

The two-tone paradigm is also often used to probe the phenomenon of buildup of streaming [8, 89]. The buildup highlights that streaming is a dynamic process, whereby the segregation of the two notes into separate streams is not instantaneous; but builds-up over time taking up to several seconds to emerge. In a study by Micheyl et al. [90], buildup was assessed using a variation of the two-tone paradigm using tone triplets (*ABA* − *ABA*), as shown in Fig 3B-left. Fig 3B-middle replicates results from this study [90] whereby listeners *continuously* report perception of one or two streams for different frequency separations Δ*F*. The behavioral data shows that when the frequency separation Δ*F* is large, both *A* and *B* tones are perceived as segregated streams relatively quickly. As Δ*F* decreases, the segregated percept takes longer to emerge lasting over many seconds. Fig 3B-right replicates the same behavior using the model and shows that the sequences gradually segregate into separate streams with different time constants. The model faithfully replicates human performance; demonstrating a faster buildup at large Δ*F*, slower buildup at intermediate Δ*F*, and no buildup at very small Δ*F*.

#### Complex tones

Next, we explore stream segregation using complex tones. These complexes highlight the wide range of acoustic cues that aid in the segregation of auditory scenes; including frequency separation (as shown earlier), as well as amplitude modulations (AM), harmonicity, temporal synchrony, etc. [3, 51, 58, 91]. In this simulation, we focus on the role of modulation cues in stream segregation by replicating a classic study by Grimault et al. [92] where alternating noise bursts with different AM rates are presented (Fig 3C-left). As the difference in modulation rate Δ*AM* increases, noise bursts tend to segregate into two streams with distinct AM rates. Once the rate difference Δ*AM* reaches about 2 octaves, the modulated noises fully segregate into two distinct streams. Fig 3C-middle shows human perception of segregated streams as a function of Δ*AM* replicating the results from the study by [92]; while Fig 3C-right shows the performance of the model on the same stimuli. As shown in the Figure, the model closely replicates human perception as reflected by increase in probability of stream segregation. The model appears to leverage the explicit encoding of amplitude information in its trained layers to facilitate the segregation of noise sequences into corresponding streams.

Next, we examine the role of harmonicity and temporal synchrony as putative grouping cues. Both these cues are believed to exert strong grouping, acting as a bond that fuses sound elements together as shown in a study by Micheyl et al. [93]. In this work, a target tone at frequency 1000 Hz is masked by background tones that are either harmonically related or in temporal synchrony with the the target tone. The study examines two kinds of stimuli: ‘MBS’ -multiple burst same- stimuli (Fig 3D-left) have the same burst of tones presented every time; while ‘MBD’ -multiple burst different- stimuli (Fig 3E-left) vary the harmonicity relationship between target and background tones at every burst based on different fundamental frequencies (see Methods for more details about the stimuli). Fig [3D] and [3E]-middle replicate the results from the study by Micheyl et al. [93] in which listeners detect a change in the final burst of the target tone. The study shows that when target and background tones are either harmonically related or in temporal synchrony with each other, d’ is low indicating a strong background-target fusion. Listeners’ ability to segregate the target improves when either harmonicity or sychrony is perturbed. Fig [3D] and [3E]-right show the model performance on the same MBS and MBD stimuli respectively. When target and background tones are harmonically-related or in synchrony, the model favors fusion and results in a small d’. In contrast, when perturbing harmonicity by shifting the harmonics, the model favors a segregated interpretation resulting in increased d’. Similarly, when target and background tones are asynchronous, there is a significant increase in d’, again suggesting strong segregation.

#### Speech intelligibility

Next, we examine the model’s behavior using complex sounds such as speech in presence of competing noise. In all experiments, a speech utterance is presented to the network either in clean or masked by background noise that includes speech modulated noise, babble noise, cafe noise or an interfering speech utterance. All speech utterances are part of the CRM corpus where each utterance consists of a call sign and a color–number combination, all embedded in a carrier phrase [94]. A typical sentence would be “Ready baron, go to red four now,” where ‘baron’ is the call sign, and ‘red’-‘four’ is the color–number combination. Fig [3F] and [3G]-left show spectrograms of speech utterances from the CRM corpus mixed with speech modulated noise and an interfering speech utterance respectively.

Fig [3F] and [3G]-middle replicate the results from two behavioral studies using the CRM corpus in a dichotic listening paradigm where subjects identified the “number” and “color” mentioned in the target utterance under different noise conditions [95, 96]. The behavioral data yield a measure of speech intelligibility (in word percent correct) as a function of signal to noise ratio (SNR) with different noise maskers. Fig [3F] and [3G]-right depict the model’s performance replicating the same paradigm as closely as possible (see Methods for details). The model yields a correct identification of speech tokens (numbers, colors, or both) that is closely related to the SNR condition following an S-shaped curve typical of similar measures of speech intelligibility in noise. The model performance plateaus at about 98% correct identification at SNRs above 3dB (Fig 3F-right); whereas it degrades quite rapidly from -3 to -9 dB before reaching chance performance at -18 dB. When comparing effects of noise type, both human and model performance are poorer in presence of an interfering utterance, relative to babble and cafe noise conditions.

### Model function and malfunction

As outlined earlier, Fig 3 contrasts the model’s performance against reported human perceptual results in a range of stream segregation experiments. Next, we reexamine our initial hypotheses; namely that the model is able to infer simultaneous and sequential grouping cues by learning statistical regularities in natural soundscapes. The experimental results shown in the previous section suggest that simultaneous cues (tonotopic organization, AM rate, harmonicity, temporal synchrony, etc), sequential cues and grouping mechanisms play an important role in streaming paradigms. In order to shed light on their individual contributions, we run a series of *control* experiments where we look at malfunctions in the model if certain components of the system are disrupted individually.

#### Role of simultaneous cues

**Fig 4:**
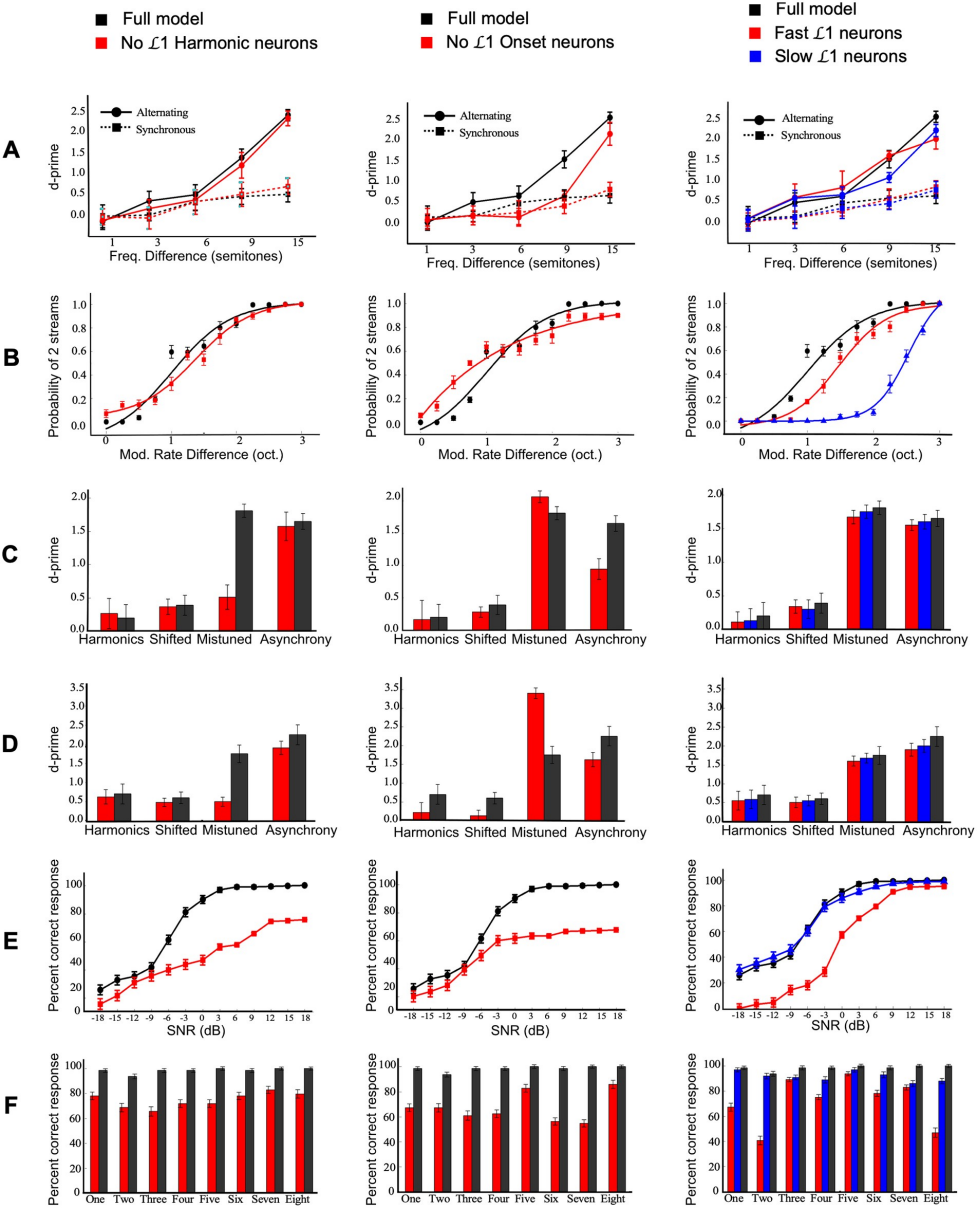
Control experiments introducing malfunction in layer $L_{1}$. The layout of the figure in each column is similar to that of Fig 3 showing model response to different stimuli. The leftmost column remove $L_{1}$-harmonic neurons, middle column removes $L_{1}$-onset neurons and rightmost column contrasts only fast or slow $L_{1}$ neurons.

The tuning characteristics of layer $L_{1}$ show that model neurons naturally cluster around specific modulation regions, hence, revealing a wide selectivity to different acoustic cues that emerge in natural sounds. Here, we focus on four $L_{1}$ neuron clusters with particular selectivity to harmonicity, onsets, fast and slow temporal modulations. We individually ‘turn off’ each of these clusters from the system and replicate all stream segregation experiments shown earlier. Fig 4 shows the model performance as follows: The leftmost column shows the model performance when $L_{1}$ harmonicity-neurons are turned off, the middle column with $L_{1}$ onset neurons turned off, and the rightmost column with fast and slow $L_{1}$ units turned off respectively. In these experiments, $L_{2}$ is not altered but is retrained based on a modified input (i.e. its input dimensionality is reduced because harmonicity, onset, slow or fast channels are removed).

Switching off harmonicity-$L_{1}$ nodes has no effect on the system’s performance in a two tone paradigm (Fig 4A-left) or sinusoidally amplitude-modulated noise bursts (Fig 4B-left). In contrast, the ability to segregate MBS and MBD sequences in case of mistuned harmonics is drastically affected by the absence of harmonicity-tuned nodes in the network (Fig 4C and 4D-left). Similarly, the network’s ability to detect speech (both colors and numbers in the CRM corpus) is severely impacted in absence of harmonicity-tuned nodes (Fig 4E-left). Taking a closer look at the behavior of the network in detecting numbers, we note a systematic drop in performance across all digits which all contain prominent voiced phonemes (Fig 4F-left).

Disabling $L_{1}$-onset nodes results in its own malfunction of the model. Streaming two-tone sequences and sinusoidally amplitude-modulated noise bursts is not affected by switching off onset units (Fig 4A and 4B-middle). However, the MBD and MBS stimuli appear to be affected in an interesting way (Fig 4c and 4D-middle) where we note an improvement of segregation in case of mistuned harmonics. The design of these stimuli puts temporal synchrony and harmonicity in conflict. Free of onset-detectors, the model is able to judge segregation mostly driven by harmonicity or lack thereof in the case of mistuning. Conversely, in case of temporal asynchrony, there is a drop in segregation performance in absence of onset-detectors, though the model is able to exploit the harmonic relationship between target and background tones to induce streaming. A comparable drop in speech intelligibility performance is also noted (Fig 4E-middle), attesting to the important role of onsets in speech perception. Taking a closer look at the model performance with individual digits (Fig 4F-middle), we note severe drops for tokens like “three”, “six” and “seven” that contain prominent fricative and plosive unvoiced phonemes.

Selectivity to temporal dynamics plays a complementary role in the model’s ability to perform stream segregation. We manipulate the selectivity of $L_{1}$ neurons to different range of amplitude modulations by testing only-slow (< 25 Hz) or only-fast neurons (> 25 Hz). The segregation of two-tone sequences appears to be unaffected by presence or absence of slow or fast units alone, and is likely mostly driven by the tonotopic organization of the nodes in the network (Fig 4A-right). In contrast, streaming of sinusoidally-modulated noise bursts is heavily affected when $L_{1}$ units tuned to faster modulations are turned off, though only mild changes are noted when slower-units are turned off (Fig 4B-right). Streaming of MBD and MBS sequences appears unaffected by the time-constants of temporal modulations left in the $L_{1}$ layer; and we observe no changes to the model behavior (Fig 4C and 4D-right). Interestingly, speech intelligibility is also unaffected when faster $L_{1}$ units are turned off (Fig 4E-right). In contrast, switching off slower units drastically affects the model’s ability to separate speech from noise, especially at low SNRs, strongly corroborating the role of midrange-modulations in speech perception [76].

#### Role of sequential temporal dynamics

**Fig 5:**
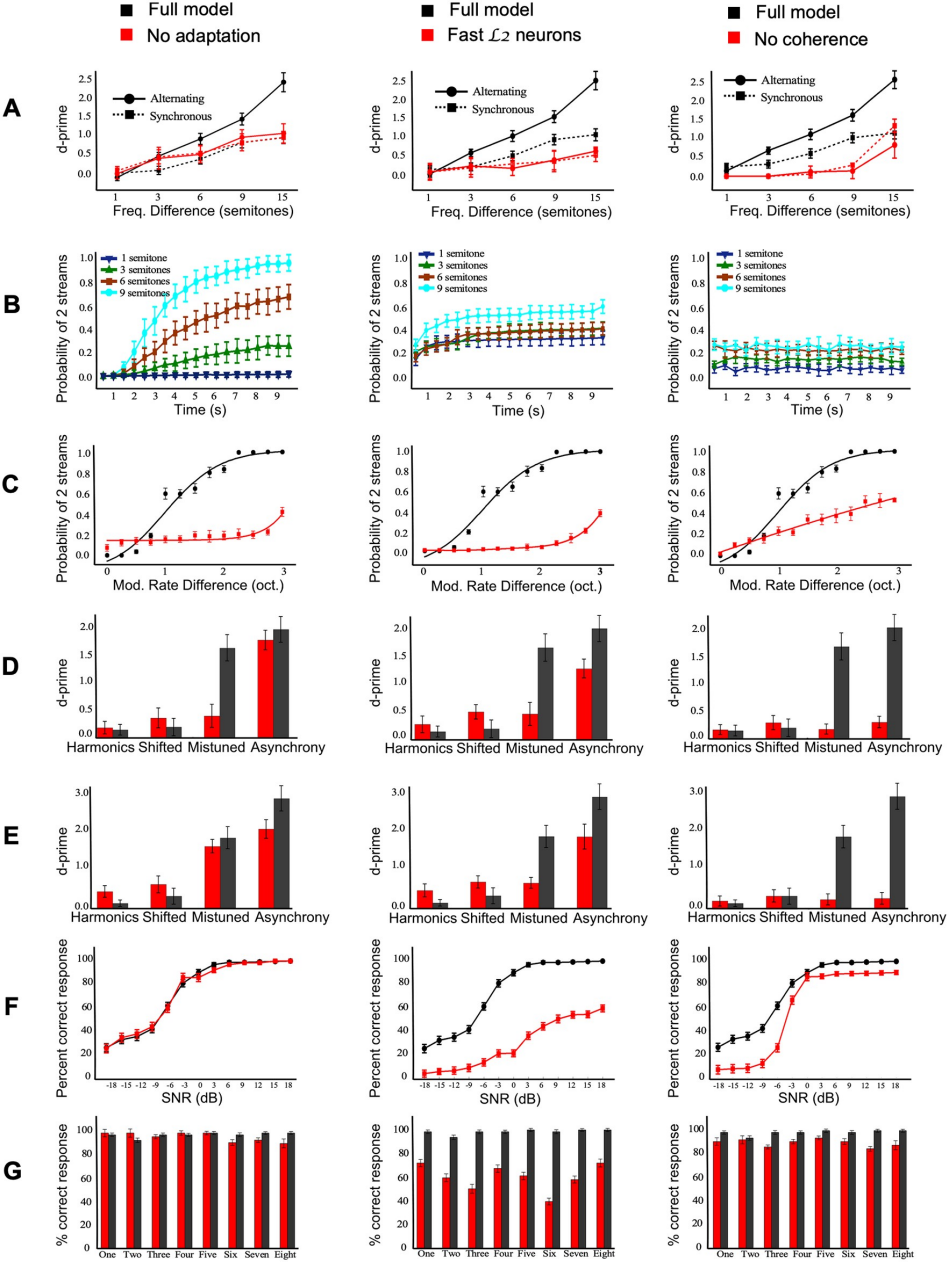
Control experiments introducing malfunction in temporal dynamics of the network. The layout of the figure in each column is similar to that of Fig 3 showing model response to different stimuli. The leftmost column remove neural adaptation, middle column removes $L_{2}$-slow neurons and rightmost column removes the $L_{3}$ temporal coherence.

Next, we examine the impact of model parameters responsible for temporal integration on stream segregation over longer time scales. First, we observe the model’s behavior if we switch off neural adaptation at the output of $L_{1}$ nodes. This mechanism aims to adjust the dynamics of neurons’ responses by eliminating nodes with moderate activation over time. Fig 5-left contrasts the model’s performance with and without this neural adaptation. Fig 5A-left shows that neural adaptation is important for segregating alternating two-tone sequences. Adaptation appears to aid the temporal coherence layer in ‘shutting down’ neurons from competing streams which facilitates segregation. In its absence, both tones in the stimulus continue to compete at the output of the model hence affecting the ability to segregate. Furthermore, this continued competition appears to slow-down the buildup process (Fig 5B-left compared to the original model behavior in Fig 5B-right). As noted in the figure, a tone sequence with frequency separation of Δ*F* = 9 semitones takes many seconds to eventually reach a segregated percept with modified model as compared to 1-2 secs in the original model, owing to the continued competition between the two tones. While the temporal coherence model is able to note the out-phase relationship between the streams, this process is assisted by neural adaptation which supresses activity from competing streams hence speeding up stream segregation in line with observed behavioral responses (Fig 3B-middle). A similar behavior is observed in case of sinusoidally amplitude-modulated noise bursts in Fig 5C-left. Here again, removing adaptation from the network allows competition across channels to linger longer hence hampering the role of temporal coherence in detecting consistent incoherent activity across competing streams. In the case of MBD and MBS sequences, adaptation appears to have a mild effect with the exception of mistuned harmonics in the case of MBD sequences and temporal asynchrony for MBS sequences (Fig 5D and 5E-left).

We next explore the role of temporal dynamics in cue extraction, particularly the role of slower time-constants which are thought to play a crucial role in sequential integration of acoustic cues as the scene evolves. We probe this role in a control experiment by switching off the $L_{2}$ units with strong selectivity to very slow modulation rates (< 15 Hz) and compare this modified network against the full architecture. The results comparing the two models are shown in Fig 5-middle and reveal wide spread after-effects across all streaming experiments. In the case of the two-tone paradigm, removing slower neurons from $L_{2}$ significantly impairs the network’s ability to segregate 2 streams as Δ*F* increases (Fig 5A-middle). Also of note is that the streaming buildup is severely affected and quickly settles on final assessment of segregation between streams regardless of Δ*F* value likely reflecting the inherent spectral-based separation across the neurons in the network but failing to track how activity across the neural population evolves over time (Fig 5B-middle). Segregation of modulated noise bursts is also severely affected (Fig 5C-middle). The probability of perceiving 2 streams drops dramatically, indicating a poor integration of neural activity across differentiated neurons. The same effect is observed in the case of MBS and MBD sequences, where the network fails to segregate the target tone from background masker tones even in presence of mistuned harmonic relationships (Fig 5D and 5E-middle). This drop is also noted for both stimuli in the case of asynchrony, even though the drop is not as dramatic, suggesting the network still relied on some degree of temporal alignment across the fast neurons remaining in the $L_{2}$ network to judge relationship between tone bursts. Finally, in the case of speech in noise experiments, the network containing ‘faster’ neurons is severely impaired across all SNR values (Fig 5F-middle). The drop in performance is clear across all digits (Fig 5G-middle). The absence of slow $L_{2}$ units clearly affects the network’s ability to match the slow changes in temporal structure of speech tokens even in presence of simultaneous cues hence failing to facilitate stream segregation. This result reinforces the joint role of both spectral and temporal (local and global) attributes in speech encoding and comprehension [76, 97].

Finally, the role of temporal fusion across channels is examined by testing the model’s performance without the temporal coherence mechanism in layer $L_{3}$. Much like earlier control experiments, removing temporal coherence has sweeping effects on the model’s ability to perform stream segregation. In the two-tone paradigm, the model treats the synchronous and alternating notes similarly as it fails to judge the phase relationship across spectral channels (Fig 5A-right). The buildup of streaming is also completely annihilated regardless of frequency separation across channels strongly suggesting that integration over time and across frequency channels plays an important role in the brain’s ability to consolidate information spectrally and temporally while it examines possible configurations or interpretations of the scene (Fig 5B-right). This process is very much what the temporal coherence stage contributes and is clearly impaired without coherence. Segregation of modulated noise bursts is also affected although the probability of segregation does increase with increased AM rate difference Δ*AM* albeit with reduced probability suggesting poorer segregation performance of the modified network (Fig 5C-right). In the case of noise complexes in the MBD and MBS paradigm, the network completely fails to achieve any form of segregation (Fig 5D and 5E-right) suggesting that the presence of simultaneous cues (e.g. harmonicity) is not sufficient. Complex noise patterns tend to activate a wide range of channels which require an integration mechanism such as $L_{3}$ temporal coherence to interpret the across-channel consistency and phase relationships. Speech segregation is slightly affected by disabling temporal coherence (Fig 5F-right) and more noticeably at lower SNR values for both colors and numbers. Fig 5G-right) highlights these mild reductions in segregation that are observed consistently across all digits.

## Discussion

The current study presents a biologically-plausible model of stream segregation that leverages the multiplexed and non-linear representation of sounds along an auditory hierarchy. While the model is formulated to focus on local and global cues in everyday sounds, it is structured to ‘learn’ these cues directly from the data. The unsupervised nature of the architecture yields physiologically and perceptually meaningful tuning of model neurons that support the organization of sounds into distinct auditory objects. The three key components of the architecture as shown in Fig 1 are: (1) A stochastic network *RBM layer* that encodes two-dimensional input spectrogram into *localized* specto-temporal bases based on short term feature analysis; (2) A dynamic *cRBM layer* that captures the long-term temporal dependencies across spectro-temporal bases characterizing the transformation of sound from fast changing details to slower dynamics. (3) A *temporal coherence layer* that mimics the hebbian process of binding local and global details together to mediate the mapping from feature space to formation of auditory objects.

The layout of the model closely replicates the physiological layout of auditory processing in the brain where an acoustic signal undergoes a series of transformations from the cochlea all the way to auditory cortex (A1), effectively extracting a rich feature representation that forms the basis for perceptual grouping of sound objects [98–103]. The sound transformations in the biological system evolve in temporal and spectral resolutions from temporally fast, spectrally refined as is typically observed in the periphery and levels of the midbrain to markedly slower and spectrally broader in cortical networks [81, 104, 105]. The current model ‘learns’ similar structures as can be seen from the modulation transfer functions for both layers $L_{1}$ and $L_{2}$ (Fig 2). The fact that the model evolves in temporal resolution from short to longer analyses is not surprising as it is one of the structural designs of the system. However, the detailed analyses learned in each layer are intriguing and suggest a close connection between neural selectivity along the auditory pathway and the progression of processes underlying Gestalt principles from a local analysis of simultaneous cues to global sequential cues [8]. This connection has in fact been postulated in a number of studies of auditory neurophysiology, particularly contrasting the differences in tuning characteristics between individual neurons in the midbrain (particularly the inferior colliculus) and cortex [81, 106, 107]. The current model does appear to also exhibit a similar variety of tuning characteristics and it is tempting to interpret the modulation profiles emergent from layers $L_{1}$ and $L_{2}$ as potentially aligned with a midbrain/cortex hierarchy. However, we should also entertain the possibility that both layers $L_{1}$ and $L_{2}$ could map to different sub-populations in auditory cortex. Cortical substructures have been reported to exhibit a variety of heterogeneous behaviors and variability in encoding temporal details about an incoming sound by multiplexing temporal and rate representations [108]. Interpreting the model output based on such dichotomy in integration mechanisms raises an interesting possibility attributing statistical-constraints of Gestalt cues solely to cortical networks in the brain. This alternative merits further examination in future work especially considering more intricate network architectures that extend across more layers including extra hidden layers in a true tradition of deep learning [68]. Follow-up analyses should also examine the encoding of stimulus features across an even wider array of temporal resolutions that span the contribution of finer details including temporal fine structure to even longer multi-second time dynamics [109, 110].

*Role of simultaneous layer*. Extracting relevant information from incoming acoustic waves is the backbone of any processing and sound interpretation system. The model replicates this feature analysis in a data-driven fashion by employing a diverse dataset of natural sounds including human speech, animal vocalizations and street ambient sounds. Structuring the local layer using a RBM architecture allows the model to learn a rich tiling of spectro-temporal basis functions. The results indicate that these bases capture fine details in the acoustic stimulus, as suggested by the modulation transfer function (Fig 2). The tuning of individual model neurons is itself well-structured and localized in this spectro-temporal space with clear organization of subsets to a wide range of acoustic cues spanning frequency proximity, harmonicity, onset, and AM rate among others, as shown by the clustering analysis.

Traditionally, biomimetic computational models of stream segregation have attempted to replicate some or all of these cues to enable stream segregation. Often, this process is achieved by hand-selecting specific axes of feature analysis that best suit the auditory scenes of interest in these specific studies [111–113]. One of the drawbacks to feature selectivity in model design is confining the testable signals to those that take advantage of these specific features. By employing an unsupervised approach to feature selection, the current model not only replicates known simultaneous cues in auditory scene analysis, but also nonlinearly spans multitudes of features given the fully-connected nature of the Restricted Boltzman Machine (RBM) used in layer $L_{1}$. Across-feature integration is in line with recent findings suggesting that many auditory neurons are driven by a multitude of stimulus features [114]. This feature integration is particularly crucial in case of complex sounds where a multitude of dimensions provide the perceptual system with converging evidence about the organization of the scene [115, 116]. The complementary value of this cross-feature mapping is clearly visible in control experiments where dropping different components of the simultaneous layer have different effects on the model’s ability to perform stream segregation (Fig 4).

*Role of sequential layer*. Along the same lines, the sequential layer provides an integrated non-linear mapping of the feature space from localized details to slowly evolving spectro-temporal patterns. The use of a cRBM layer allows the model to ‘learn’ tuning from natural sounds along slower time-constants. The transfer function analysis reveals a strong selectivity to slow temporal modulations present in natural sounds typically in the range ∼ 2–32 Hz as shown in Fig 2. This tuning is reminiscent of modulation transfer functions derived from the mammalian auditory cortex revealing neurons that are slightly broader spectrally and slower temporally [81, 82, 104]. This global analysis has not been extensively investigated in models of auditory scene analysis, though few models have leveraged cortical-like processing to complement local feature analysis [113, 117–119]. Engineering approaches have also leveraged this global analysis especially in the case of speech processing systems. Approaches such as RASTA (relative spectra), high-pass and band-pass filtered modulation spectra take advantage of slow articulatory structures of speech production as well as the sensitivity of human perception to such slow dynamics to offer a more robust processing of speech sounds in presence of noise [120–122].

*Role of temporal coherence layer*. While feature analysis is a crucial ingredient in auditory scene analysis, fusing the relevant cues together is an equally important complementary stage to group the features into meaningful objects. Perceptual and physiological data have strongly suggested that temporal coherence achieves the feature fusion needed for object formation [64, 123, 124]. While its exact neural underpinnings are not well understood yet, empirical evidence strongly suggest that it plays an important role in scene organization by the auditory system [39, 65, 125, 126]. Indirect neurophysiological evidence suggest that coherence mechanisms operate beyond auditory cortex likely in a network engaging the intraparietal sulcus and superior temporal sulcus [65, 126–128]. The current model employs a rather simple biologically-plausible Hebbian interaction across channels to rapidly adapt co-operative and competitive interactions between coherent and non-coherent responses [72]. Effectively, channels that exhibit a high degree of temporal correlation across feature dynamics are mutually strengthened while incoherent channels are gradually weakened hence facilitating segregation of target signals from background interference. Naturally, the Hebbian-based approach is not the only implementation for this fusion stage and numerous techniques for such fusion have been explored in areas of data mining and analytics. In fact, feature fusion has become an important topic of research in the deep learning literature particularly when applied to computer vision and sensor networks. Ultimately, the architecture used to implement such grouping stage will have to infer relationships between activities of model sub-components based on some pre-defined loss function (in case of unsupervised learning). In the current model, we reduced this learning function to the basic principle of temporal coherence [64].

### Scene segregation and fusion

The analysis of control experiments quantifies the complementarity of rich feature representation and grouping mechanisms in driving scene segregation. The proposed architecture faithfully replicates human psychoacoustic behavior on steaming paradigms over wide range of stimuli ranging from simple tones to speech utterances as demonstrated in Fig 3. In case of two tone streaming paradigm shown in (Fig 3A), the network exhibits stream segregation when two alternating tones are widely separated across tonotopic frequency axis. This behavior is consistent with well established psychophysical and physiological findings of stream segregation induced by differences in tonotopic cues [129–132]; and relies heavily on the activation of different groups of neurons with distinct frequency selectivities as captured in $L_{1}$. In absence of temporal correlation between these two groups, the temporal coherence layer aided by the adaptation mechanism suppresses the anti-correlated groups of units, hence inducing stream segregation in the final stage of the network. However when Δ*F* is small enough, there is high degree of overlap resulting in a single stream percept. This segregation/integration effect is strongly maintained regardless of a number of manipulations to the model architecture. The key components crucial to the organization of tone sequences are the presence of tonotopic or frequency selectivity combined with temporal integration that examines activity across neural channels at relatively longer time-scales. This observation is very much in line with the spatio-temporal view of auditory stream segregation which requires neural channels to be widely separated in addition to temporal asynchrony across these channels [133].

The interaction of spectral and temporal dynamics during the organization of tone sequences supports the view of stream segregation as a dynamic process. The buildup effect reported in the current model (Fig 3B) is in line with established psychoacoustic behaviors [90, 134–136] and suggests that segregation of two streams is not instantaneous; but strengthens over time and can lead to segregation when frequency difference (Δ*F*) is large enough. The current model highlights that this effect is in fact reflecting the competition across neural channels as viewed by the temporal coherence layer. The binding of correlated groups of neurons strengthens over time while suppressing the anti-correlated units over time in the same process. Interaction across multiple features is also noted in other simulations that pit against each other harmonicity, onsets and temporal dynamics (Fig 3[C], 3[D] and 3[E]). Simulations using complex tones directly examine the role of localized spectro-temporal tuning in $L_{1}$ as an encoding of simultaneous cues such as harmonicity, onset and fast amplitude modulations among others. Sequential cues emergent in $L_{2}$ are crucial in tracking the activity emerging in the localized layer over longer amplitude modulations; which are then fused together in the last $L_{3}$ layer.

Through this rich selectivity learned directly from natural sounds, the network offers a wide span of selectivity across the spectrotemporal space. This tuning proves effective in tackling complex auditory scenes composed of speech with various interferers. In line with human perceptual data, the model shows that speech tokens are harder to identify in presence of utterances from same corpus compared to babble and cafe noise as the signal-to-noise ratio gets smaller. The model highlights that this variable response is largely caused by the dominance of neural activity from the interfering set relative to the target. The distinct activation between target and interferer is further blurred in absence of of slow sequential cues which integrate information about the speech utterance beyond just that target number/color. As shown in the control experiments, a network that lacks slow sequential cues is further impaired in making a judgment about the identity of the target token, likely due a to an enhanced confusion between its representation and that of the interferer. Once this activity reaches the temporal coherence layer, the weakly responsive neurons get suppressed, hence resulting in the actual number/color token getting wrongly identified as the one in the interfering utterance.

### Concluding remarks

Overall, the proposed model highlights three key results: (i) Using the right configuration, we are able to infer a wide-range of Gestalt cues directly from natural sounds. The proposed RBM architecture offers a cooperative and nonlinear integration of these cues to result in a multiplexed representation of auditory scenes across various granularities in time and frequency. By using an unsupervised learning approach, the network is not being optimized for a specific application; rather, it is reflecting the inherent variety of local and global dynamics present in natural sounds. Possibly, an even deeper neural architecture extending beyond just a few layers could extend the rich feature analysis and fill in the spectrum from local to global hence adding a more refined mapping along with the nonlinear integration naturally offered by the RBM architecture. (ii) Grouping acoustic features is effectively an outlook across *all* active nodes that allows to piece together the pieces of each auditory object. This process effectively plays 2 key roles: a grouping role by putting together pieces of a sound object (effectively integrating together pitch, timbre, rhythm and possibly space information that reflect a common object); and an elimination role by suppressing channels that are irrelevant to the emergence of the foreground object, hence enhancing the signal-to-noise ratio in the network. Temporal coherence is one such fusion mechanism that has been garnering stronger neural and perceptual evidence [39, 65, 125, 126]. The current work employs Hebbian learning, a biological simple mechanism that affords such fusion over the rightly chosen time-scales. (iii) Auditory scene segregation is a balancing act of the proper feature analysis along with mechanisms for fusion that give rise of auditory object representations. While both stages are necessary, neither one is sufficient. The proposed model offers a unified platform that integrates together these different mechanisms and strategies. It also bridges the existing physiological theories of scene organization with perceptual accounts of auditory scene analysis.

## Materials and methods

### Network architecture

The proposed model is structured along 4 key stages: initial data pre-processing by transforming the acoustic signal to a time-frequency representation, a local analysis over short time-scales, a global analysis over an array of longer time-scales, then a fusion stage using temporal coherence. A final readout of the network activity is implemented to extract information from specific streaming experiments to probe segregation of individual streams in the input scene. Details of each component of the model are outlined next:

The acoustic signal is first analyzed through a model of peripheral processing in the mammalian auditory system, following the model by Yang et al. [66]. Briefly, it transforms the acoustic stimulus sampled at 8KHz into a joint time-frequency representation referred to as auditory spectrogram. The stage starts with a bank of 128 asymmetric constant-Q filters equally-spaced on a logarithmic axis over 5.3 octaves spanning the range 180 Hz to 4000 Hz (*Q*_*ERB*_ ≈ 4) [137]. By its very nature, the peripheral model uses a non-parametric set of cochlear filters that are fixed over a span of 5.3 octaves (see [66] for details). In the current model, we cap our sampling rate to 8KHz in order to provide ample coverage over lower frequency regions. After cochlear filtering, the outputs undergo spectral sharpening via first order derivative along frequency, followed by half-wave rectification then short term integration with *e*^−*t*/*τ*^ where *τ* = 10 ms. This filterbank analysis results in a time-frequency auditory spectrogram represented by *S*(*t*, *f*). Three consecutive frames are then grouped together to form a one dimensional vector *x* such that *x* ∈ *R*^*n*^ and *n* = 384. This process is repeated for all the audio samples in the dataset to form a set of *N* sampled patches given by *X* = *x*^1^, *x*^2^, …, *x*^*N*^. This set of time-frequency patches (*X*) constitutes the input to second component of the network.

#### Simultaneous layer

The simultaneous layer $L_{1}$ is structured as a Sparse Restricted Boltzmann machines (RBM), which is chosen to discover features from an unlabeled set in an unsupervised fashion [67, 138]. Sparse RBMs are undirected graphical models with *K* binary hidden variables. The energy function of a RBM is defined as:

$$\begin{array}{c} \begin{array}{c} E\left(x,h\right)=\frac{1}{2}\sum_{k}{\left(x_{k}-A_{k}\right)}^{2}-\sum_{l}B_{l}h_{l}-\sum_{k,l}x_{k}h_{l}w_{kl} \end{array} \end{array}$$ (1)

where, *x*_*k*_ and *h*_*l*_ denote the states of *k*^*th*^ visible unit and *l*^*th*^ hidden unit, while *w*_*kl*_ represents the strength of connection between them and *A* (and *B*) are the visible (and hidden) biases, respectively. The joint energy distribution of (*x*, *h*) is defined as:

$$\begin{array}{c} \begin{array}{c} P\left(x,h\right)=\frac{1}{Z}exp\left\{-E\left(x,h\right)\right\} \end{array} \end{array}$$ (2)

$$\begin{array}{c} \begin{array}{c} Z=\sum_{x,h}exp\left\{-E\left(x,h\right)\right\} \end{array} \end{array}$$ (3)

where, *Z* is a normalizing partition function which is obtained by summing the energy function *E*(*x*, *h*) over all possible combinations of visible and hidden units.

Given observed data, the states of hidden units are conditionally independent. Their activation probabilities are,

$$\begin{array}{c} \begin{array}{c} P\left(h_{j}|x\right)=\frac{1}{1+exp\{-x^{T}w_{l}\}} \end{array} \end{array}$$ (4)

where *w*_*l*_ denotes the *l*^*th*^ column of *W* and represents connection weights between the *l*^*th*^ hidden unit and all visible units. We incorporate sparsity into the hidden layer representation to ensure that hidden activations are more selective to specific characteristics of the training data. A sparsity penalty is imposed on the activation of hidden units such that the probability of a hidden unit being active, denoted by *q* should be as close as possible to a specified “sparsity target”, given by *p*. The penalty term is chosen to be the cross entropy between the desired and actual distributions given by: *p* log *q* + (1 − *p*) log(1 − *q*) [68]. The measure imposes a ‘sparsity-cost’ that allows to adjust both the bias and weights of each hidden unit in the network.

Given an input signal, a hidden unit is said to be *representing* a particular data sample when it is activated. The objective of generative training of RBMs is to maximize the marginal distribution of visible units *P*(*x*) which is typically done using *Contrastive Divergence* (CD) [69, 139]. This algorithm updates the feature of the *k*-th hidden unit seeing the training data *x*_*l*_ such that:

$$\begin{array}{c} \begin{array}{c} \Delta w_{k}=P\left(h_{k}=1|x_{\left(l\right)}\right)\cdot x_{\left(l\right)}-P\left(h_{k}=1|x_{(l)-}\right)\cdot x_{(l)-} \end{array} \end{array}$$ (5)

where *x*_(*l*)−_ is sampled from *P*(*x*|*h*_*l*_). The algorithm learns the distribution of hidden activations *h*_*k*_ such that *x*_(*l*)−_ -when sampled from the hidden activations- come close to the real distribution of visible units *x*. As hidden activations *h*_*k*_ keep on learning the representation of visible units *x*, the update rule Δ*w*_*k*_ keeps decreasing. The learning process only stops when the reconstruction is close to perfect i.e. (*x*_(*l*)_ − *x*_(*l*)−_) approaches 0.

The model used here employs 400 hidden units in the simultaneous layer $L_{1}$. Once trained, the weights *W* yield unique spectro-temporal basis functions. We then transform the weights *W* into two-dimensional functions F(t,f) where *t* denotes a patch of 30 ms and *f* corresponds to the frequency axis of auditory spectrogram. These 2D filters are then applied in a convolutional fashion onto the time-frequency patch *S*(*t*, *f*) to obtain the filter response ${\hat{r}}_{k}\left(t\right)$ given by:

$$\begin{array}{c} {\hat{r}}_{k}\left(t\right)=\sum_{f}\int S_{l}\left(\tau ,f\right)F\left(t-\tau ,f\right)d\tau \end{array}$$ (6)

These responses $\left\{{\hat{r}}_{k}\left(t\right)\right\}$ then undergo an adaptation process that allows to strengthen the contrast between foreground and background units. This mechanism follows a classic closed-loop synaptic adaptation proposed by Tsodyks et al. [140] given by:

$$\begin{array}{c} \frac{\delta a(t)}{dt}=\frac{1-a(t)}{\tau_{a}}-\alpha a\left(t\right){\hat{r}}_{k}\left(t\right) \end{array}$$ (7)

$$\begin{array}{c} r_{k}\left(t\right)=a\left(t\right){\hat{r}}_{k}\left(t\right) \end{array}$$ (8)

with time constant *τ*_*a*_ = 300 ms and synaptic utilization parameter *α* = 1*e*^−5^. This operation yields output responses {**r**_*k*_(*t*)} that are then processed through the next layer in the hierarchy. A range of other adaptation parameters *τ*_*a*_ and *α* (around the chosen values) were explored with qualitatively similar results.

#### Sequential layer

The next layer $L_{2}$ is structured as an array of conditional RBMs (cRBM) [71]. cRBMs are non-linear generative models for time series data that employ undirected models with visible units {*x*_*k*_} connected to a layer of binary latent variables {*h*_*k*_}. In the present model, the visible units {*x*_*k*_} are represented by a Gaussian distribution fitted over $L_{1}$ responses. At each time step *t*, the model maintains a history of the last *τ* time steps and stores the visible variables corresponding to these time steps in a *history* vector referred to as *x*_*τ*_. Each visible input {*x*_*k*_} and hidden unit {*h*_*k*_} at a particular time step *t* receives directed connections from the history vector *x*_*τ*_ so as to capture long term temporal dependencies across visible units. This dynamical model is defined by a joint distribution:

$$\begin{array}{c} P\left(x\left(t\right),h\left(t\right)|x_{\tau }\right)=exp\left\{-E\left(x\left(t\right),h\left(t\right)|x_{\tau }\right)\right\}/Z\left(x_{\tau }\right) \end{array}$$ (9)

where *x*(*t*) is a Gaussian fitted representation of $L_{1}$ filter responses over time, *h*(*t*) is a collection of binary hidden units such that *h*(*t*) ∈ (0, 1), *x*_*τ*_ contains the history of past *τ* filter responses, and *Z* is the partition function as explained in the previous section. The energy function ***E*** is given by:

$$\begin{array}{c} \begin{array}{c} E\left(x\left(t\right),h\left(t\right)|x_{\tau }\right)=\frac{1}{2}\sum_{k}{\left(x_{k}\left(t\right)-{\hat{a}}_{k}\left(t\right)\right)}^{2}-\sum_{l}h_{l}\left(t\right){\hat{b}}_{l}\left(t\right)-\sum_{k,l}W_{kl}x_{k}\left(t\right)h_{l}\left(t\right) \end{array} \end{array}$$ (10)

where *W* captures the connections between input and hidden variables. The dynamical terms ${\hat{a}}_{k}\left(t\right)$ and ${\hat{b}}_{l}\left(t\right)$ are linear functions of previous *τ* filter responses *x*_*τ*_, given by:

$$\begin{array}{c} \begin{array}{c} {\hat{a}}_{k}\left(t\right)=(A_{k}+\sum_{m}C_{km}x_{mK}\left(t\right))\;{\hat{b}}_{l}\left(t\right)=(B_{l}+\sum_{m}D_{lm}h_{mK}\left(t\right)) \end{array} \end{array}$$ (11)

where *A* and *B* are static biases and *C* and *D* are autoregressive model parameters. The dynamic biases $\hat{a}$ and $\hat{b}$ integrate the input over past *τ* time steps and apply them as a bias to the visible unit *x*_*k*_(*t*) and hidden unit *h*_*l*_(*t*) at current time step *t*. The parameter set Θ = {*W*, *A*^*τ*^, *B*^*τ*^, *C*^*τ*^, *D*^*τ*^} of cRBM networks are learned using contrastive divergence (CD) similar to layer $L_{1}$ [69, 139].

Layer $L_{2}$ is structured as an array of cRBM networks spanning various time histories. In the current model, we define networks with time constants *τ* ranging between 30–600 ms. For each time constant, a matching number of instances of layer $L_{1}$ responses are grouped and analyzed in parallel. The same training data (as outlined later) is used to train the RBM in layer $L_{1}$ as well as the cRBM in $L_{2}$, though training occurs individually for each layer. Here, we employ 300 nodes for each layer of each cRBM network.

#### Temporal coherence layer

The activations from layer $L_{2}$ are further processed using a Hebbian network, which implement a Storkey learning rule [74], written as:

$$\begin{array}{c} \begin{array}{c} v_{ij}\left(t\right)=v_{ij}\left(t-1\right)+r_{i}\left(t\right)r_{j}\left(t\right) \end{array} \end{array}$$ (12)

where *v*_*ij*_(*t*) is a coherence synaptic connection weight between two neurons *i*^*th*^ and *j*^*th*^ in $L_{2}$ at time *t*, *r*_*i*_(*t*) and *r*_*j*_(*t*) are the responses of *i*^*th*^ and *j*^*th*^ $L_{2}$ neurons respectively and *v*_*ij*_(*t* − 1) is the connection weight between the same two neurons at time *t* − 1. The equation above shows that if both *r*_*i*_(*t*) and *r*_*j*_(*t*) are ‘coherent’, the synaptic connection between them becomes stronger whereas the synaptic connections gets weaker for anti-correlated responses. Given that this stage occurs after the sequential integration layer, the coherence is indeed assessed over time histories used in each cRBM network in $L_{2}$. This stage effectively applies a time-dependent Hebbian weight to the output of the model resulting in $\hat{R_{t}}=R_{t}V_{t}$.

#### Model dataset

An ensemble of natural sounds comprising of speech and natural sounds are assembled together into a single dataset. It includes speech segments from the TIMIT database [141] that include both male and female speakers, as well as various accents and styles and approximately amounts to 4 hours of data. It also comprises the BBC sound effects database [142] which contains environmental sounds like ambient and outdoor noises (e.g. street, office, warfare and transportation) as well as animal vocalizations (e.g. barking dogs, bleating goats, and chattering monkeys). The BBC database has total of 2400 recordings, amounting to 68 hours of data. All signals are analyzed over 3-sec segments. Speech utterances are approximately 3 seconds in length, while animal vocalizations and ambient sounds are broken into 3 seconds, and windowed using a raised cosine window to avoid transient effects. All segments are down sampled to 8 kHz and standardized to be zero-mean and unit variance.

### Model characterization

#### Ripple stimuli

The modulation transfer function (MTF) for each layer is characterized using ripple stimuli [143]. They are broadband noises consisting of 280 tones, equally spaced along the logarithmic frequency axis, over a range of 5 octaves. The spectral envelope of these stimuli forms sinusoids whose amplitude is modulated by an amount Δ*A* that ranges from 0 to 100%. This construction forms a drifting sinusoidally shaped spectrum along the frequency axis. The envelope of a ripple stimulus is given by:

$$\begin{array}{c} \begin{array}{c} S(t,f)=L(1+\Delta Asin(2\pi (\omega t+\Omega f)+\phi )) \end{array} \end{array}$$ (13)

where *L* denotes the overall level of the stimulus, *t* is time, and *f* is the tonotopic axis, defined as $f=log_{2}f/f_{0}$, with *f*_0_ being the lower edge of the spectrum and f the linear frequency index. *ω* is the ripple velocity (in Hz), Ω is the ripple density (in cyc/oct), and *ϕ* is phase of the ripple.

#### Measurement of modulation transfer function (MTF)

The MTF for each layer is measured using individual ripples at rate-scale (*ω*, Ω) combinations over a range of Ω = [0.25, 16] (cyc/oct), and *ω* = [−50, 50] (Hz), with negative rates denoting upward moving ripples. The MTF calculation procedure is as follows: For each combination of (*ω*_0_, Ω_0_), we generate a ripple stimulus with contrast Δ*A* = 100% and a corresponding ripple with contrast Δ*A* = 0% that provides a base level or noise floor to the model’s response. The response of units in $L_{1}$ and $L_{2}$ to each ripple pair is then obtained (note that responses obtained from the layer $L_{1}$ are used as input for layer $L_{2}$). Then, an estimate of modulation-synchronized activity *M* at exactly *ω*_0_ is obtained from each response then converted to a normalized tuning estimate, given by:

$$\begin{array}{c} 10log_{10}\frac{\left|\right|M_{100\%}\left(\omega_{0}\right){\left|\right|}^{2}}{\left|\right|M_{0\%}\left(\omega_{0}\right){\left|\right|}^{2}} \end{array}$$ (14)

#### Agglomerative clustering

We employ a hierarchical clustering to explore emergent groupings in the structure of filters in layers $L_{1}$ and $L_{2}$. The procedure follows classic clustering techniques used in data mining to partition a dataset into subsets that share some similarity [144]. We build a hierarchy from individual $L_{1}$ and $L_{2}$ filters by employing pair-wise Euclidean distance between rate-scale tuning of the filters. The agglomerative clustering approach gradually merges individual clusters together based on a distance measure (e.g. Euclidean distance). The number of clusters employed here is heuristically determined based on visual inspection of emerging groups. The two clusters of particular interest in control experiments are harmonicity and onset groups, which occupy a region centered around [1-2] cyc/oct and fast temporal modulations, respectively. We visual inspect the time-frequency profiles of each group to confirm its consistency. We also confirm that neurons grouped in the group labeled onsets (O) are indeed transient filters with an onset response (rather than offset). No apparent offset detectors emerged in the trained filters.

### Stimuli for stream segregation experiments

We test the model on stream segregation paradigms spanning tones, complexes and speech; as detailed next.

#### Two tone sequences

The two-tone stimuli consist of a sequence of repeating pure tones. The tone sequences replicate the stimulus structure used in [86] and consist of 100 ms tones, half of which are fixed at 1000 Hz, referred to as “A” tones. The other half, denoted as “B” tones, have a frequency 1, 3, 6, 9, or 15 semitones below 1000 Hz, i.e. at 943.9, 840.9, 707.1, 594.6 or 420 Hz. The A and B tones are separated by a silent gaps of 100 ms and are presented either alternately or synchronously. Each stimulus consists of a total of 24 tones, twelve A tones and twelve B tones. The total duration of sequence is 2.3 seconds for the synchronous case and 2.4 seconds for the alternating case.

*Buildup effect on stream segregation*. In order to probe streaming buildup, we use tone triplet sequences, ABA, following the stimulus paradigm used in [90]. Tone A is randomly selected from a set of 3 different frequencies (500 Hz, 1000 Hz and 2000 Hz) across different experiments and the other tone B is placed at 1, 3, 6 or 9 semitones above A in each of the experiments. Tones are 125 ms in length with no silence between triplets, though there is a silent gap of 125 ms between consecutive triplets. The buildup effect is demonstrated by varying the duration of entire stimuli sequence from 1 second to 10 seconds. The results are averaged across all the experiments and compared against the psychophysical results reported in [90].

#### Amplitude-modulated noise sequences

The stimulus paradigm used for the amplitude-modulated (AM) noise sequences closely follows the structure used in [92]. The noise sequence consists of repeating sinusoidally amplitude-modulated bursts of broadband noise, in a repeating ABA pattern, where A and B correspond to noise bursts having different modulation rates. The modulation depth is maintained at 100% throughout the experiment. Each burst is of 100 ms in duration with no silent gap in between, however there is a silent gap of 20 ms between each of the triplets. The modulation rate of A noise is kept constant at 100 Hz throughout all experiments whereas the modulation rate of B noise is varied from 100 Hz to 800 Hz across different sequences. The modulation rates of B noise are spanned such that they are 0, 0.3, 0.5, 0.7, 0.8, 1, 1.2, 1.4, 1.6, 1.8, 2, 2.5 or 3 octaves above fixed modulation rate of A noise in each sequence. The duration of all sequences is kept constant at 6.4 s.

#### Tone complexes with harmonicity and onset variations

For this experiment, we use the same stimuli sequence as used in [93]. The stimulus consists of 8 target tones denoted by A. Tone A is kept at a constant frequency of 1000 Hz throughout the sequence. Target tones are accompanied by background tones, where each of background tones are 100 ms in duration. The background tone are presented either synchronously with 100 ms targets (referred to as **sync**), or 40 ms before each 60 ms target (referred to as **async**). The offsets of the target and background tones are synchronous in all cases. In synchronous condition, we present different patterns of target and background tones along the tonotopic frequency axis, namely harmonic, shifted and mistuned condition. In “harmonic condition” (H), the background and target tones are placed harmonically in the frequency axis, where each of the tones are harmonics of fundamental frequency *f*_0_ set to be 1000/*N* and N is randomly set to 3, 4, 5, or 6, with equal probability. All harmonics with frequencies lower than 2000 Hz are included in the stimulus. According to conditions being tested, N is set to be constant for every burst in the sequence denoted by “multiple bursts same” (MBS), or is varied randomly across bursts within trial denoted by “multiple bursts different” (MBD), with the constraint that two consecutive N cannot be the same. In “shifted” condition, the bursts are constructed by shifting all the harmonics by 25% of the *f*_0_ in either direction except the target tone. In “mistuned” condition, the stimulus is generated by shifting only the target tone by 4% relative to its reference H position. In asynchronous condition, we present the target and background at specific harmonics just like the ‘H’ condition; however in this case, there is an onset difference of 40 ms between the target and background tones.

#### Speech intelligibility

This experiment replicates the paradigm used in [95, 96]. Speech sentences from multiple speakers are taken from CRM corpus [94] that contains an utterance like “Ready Baron [call sign] go to *blue* [color] *eight* [number] now”. The dataset includes four colors (blue, red, green, white) and eight numbers (1-8) in different combinations yielding 256 different sentences recorded for eight different talkers. The task is to identify the target color or number in the sentence under different SNR conditions for various noise types ranging from -18 dB to 18 dB in 3 dB steps. In order to maintain consistency with the perceptual experiments, we use speech modulated noise, babble noise, cafe noise and two-talker interferer from the actual corpus. In case of speech modulated noise, the noise signal is spectrally shaped (with a 512 point FIR filter) to match the average spectrum of 2048 sentences in the CRM corpus. The babble and cafe noise is taken from BBC sound database [142] whereas two talker interferer is taken from CRM corpus in such a way that the color and number in interferer sentences are different from target color or number.

### Readout of model segregation results

For all non-speech simulations, the final readout compares the model response to a given stimulus and to a slight variation of that stimulus in order to probe whether their respective outputs exhibit noticeable differences, which would indicate a segregated or grouped percept. Ultimately, the model readout quantifies the response difference between these signals (as a relative measure) as we sweep through the input parameters. This approach is consistent with classic techniques used to objectively probe stream segregation in human listeners (see [86] for more discussion). In the present study, a threshold is chosen empirically to quantify the difference between the stimulus and its variant in order to label it as 1 stream (small enough difference) or 2 streams (large enough difference). In all cases, we confirm that the results are qualitatively similar when we vary the choice of thresholds within a reasonable range. Details of this comparison procedure are outlined below. The procedure for segregation of speech signals is different, as specified in the speech intelligibility section.

*Two tone sequences*. In order to determine whether the tones in the ABA tone sequence are grouped into a single stream or multiple streams, we alter the last burst of the A tone by 4% of its actual frequency in either direction (upward or downward) in one sequence (represented by A’) and keep the A tone the same in another sequence. We pass both sequences through the model and compute the Euclidean distance between final responses obtained for the sequence with change and sequence with no change. As the separation between A and B tones increases, we notice that this Euclidean distance increases. We determine an empirically chosen threshold over this distance measure to indicate whether tones A and B are grouped into a single stream or form segregated streams. A d’ measure is then used to quantify correct (hit rate) and false detection (false alarm) of A for both alternating and synchronous sequence; which is computed as:

$$\begin{array}{c} d^{\prime }=z\left(H\right)-z\left(F\right) \end{array}$$ (15)

where z() represents the z-score. The d’ score determines the strength of auditory streaming, in line with the approach used in the psychophysical results reported in [86].

*Buildup effect on stream segregation*. The analysis of buildup also alters the the final burst of the sequence as either tone A or A’ as explained earlier. If the network can report any difference between A and A’ based on a thresholded Euclidean distance, we consider A as a single stream, otherwise both A and B are grouped into single stream. Here, we used the percentage of correct detection of tone A as metric to determine streaming, consistent with results reported in [90].

*Amplitude-modulated noise sequences*. In the noise sequences, the final burst is comprised of either noise A having the modulation rate of 100 Hz or noise A’ with slight alteration of 10% to actual modulation rate. The results are then reported in terms of percentage of correct detection of noise A following a similar thresholded Euclidean measure approach.

*Tone complexes with harmonicity and onset variations*. Just like previous experiments, the final burst of the sequence in each trial comprise of either target tone A or an alteration of 4% to the actual frequency of A in random direction represented by A’. A d’ analysis based on correct (hit rate) and false detection (false alarm) of A for all possible combinations is reported following the procedure described earlier.

*Speech intelligibility*. The model’s performance is assessed based on a simplified speech identification task that only employs a readout of the encoding of the target speech segments in the model. First, we divide all utterances belonging to a particular target (either number or color) into training and test sets. Each of the utterances is passed through the entire network to obtain an output response. Frames belonging to the target token are collected together and their corresponding output responses are averaged out to get a single mean response for each utterance. We collect all such responses across the entire training set and build GMM models [145] for each target. The test utterance is then passed trough the network to obtain the corresponding output response and averaged across the frames corresponding to the target token similar to training paradigm. This average response is then analyzed through each of the GMM models to obtain the log likelihood score relative to each target *P*(*target*|*θ*) where *θ* represents the GMM parameters for each target class. Based on a predetermined threshold defined empirically, a decision is made as to whether the system identifies the correct target token or not. We repeat the experiments for all the colors and numbers in the CRM corpus and report the accuracy of the system in terms of percentage correct identification of color, number and both color and number.
